# Orthopaedic publications receive fewer citations than general medicine articles: a scientometric analysis of temporal trends

**DOI:** 10.1186/s10195-026-00958-y

**Published:** 2026-07-22

**Authors:** Filippo Migliorini, Raju Vaishya, Nicola Maffulli, Jörg Eschweiler, Philipp Kobbe, Marcel Betsch, Luise Schäfer, Francesco Oliva, Fabrizio Rivera

**Affiliations:** 1 Department of Trauma and Reconstructive Surgery, University Hospital of Halle, Martin-Luther University Halle-Wittenberg, Ernst-Grube-Street 40, 06097 Halle (Saale), Germany; 2 Department of Orthopaedic and Trauma Surgery, Eifelklinik St. Brigida, Kammerbruchstr. 8, 52152 Simmerath, Germany; 3 Department of Life Sciences, Health, and Health Professions, Link Campus University, 00165 Rome, Italy; 4 Department of Orthopaedics and Joint Replacement Surgery, Indraprastha Apollo Hospital, Sarita Vihar, New Delhi, 110076 India; 5 Department of Trauma and Orthopaedic Surgery, Faculty of Medicine and Psychology, University La Sapienza, 00185 Rome, Italy; 6 School of Pharmacy and Bioengineering, Faculty of Medicine, Keele University, Stoke On Trent, ST4 7QB UK; 7 Centre for Sports and Exercise Medicine, Barts and the London School of Medicine and Dentistry, Mile End Hospital, Queen Mary University of London, London, E1 4DG UK; 8 Department of Trauma and Reconstructive Surgery, BG Klinikum Bergmannstrost Halle GmbH, Halle (Saale), Germany; 9 Department of Orthopaedic and Trauma Surgery, University Hospital Erlangen, Erlangen, Germany; 10 Department of Sports Traumatology, Università Telematica San Raffaele, Rome, Italy; 11 Department of Orthopaedics and Traumatology, Ospedale SS Annunziata, 12038 Savigliano, Italy

**Keywords:** Bibliometric, Citations, Metrics, Clarivate, Web of Science, Speciality, Impact factor

## Abstract

**Background:**

Orthopaedics, as a surgical speciality with a structurally narrower citing audience and lower citation density than general medicine, is systematically disadvantaged in any cross-disciplinary comparison relying on absolute citation counts. Whether this disadvantage is stable or has changed over time has not been previously quantified.

**Methods:**

The 100 most-cited articles indexed in the Web of Science Core Collection were identified for medicine and orthopaedics in 2014 and 2024. Citation data were extracted from a single snapshot taken on 19 January 2026. Total citations and citation velocity, defined as citations per year since publication, were compared between fields and across time points using Mann-Whitney U tests. Ratio-based analyses and distributional characteristics were also assessed.

**Results:**

Median citation counts were substantially higher in medicine than in orthopaedics in both years, reaching 5055 versus 288 in 2014 and 720.5 versus 34.0 in 2024, corresponding to 17.6- and 21.2-fold differences, respectively. Citation velocity followed the same pattern, with medicine maintaining an approximately 17- to 21-fold advantage at both time points. Within-field temporal analysis showed that citation velocity remained broadly stable in medicine between cohorts, whilst orthopaedics demonstrated reduced early citation accrual in the more recent cohort. Citation distributions were more right-skewed in medicine, with a small number of articles accounting for a disproportionate share of total citations, whereas orthopaedic citations were more evenly distributed across the top 100.

**Conclusions:**

Orthopaedics demonstrated substantially lower citation performance than general medicine articles, and this gap appeared to persist over time.

## Introduction

How good is orthopaedic research? The question sounds provocative, but it sits at the heart of a practical problem that affects careers, funding, and institutional prestige [1–3]. Citation counts have become the default currency of academic evaluation, and by that measure, orthopaedics consistently underperforms relative to general medicine [4–6]. Whether this reflects a genuine difference in scientific quality or simply a structural feature of how surgical disciplines generate and accumulate citations is a question that has been repeatedly raised in bibliometric research, yet rarely addressed with the rigour it deserves [7–9]. The reasons why orthopaedic articles attract fewer citations than medical ones are not difficult to identify. The speciality is, by nature, clinically precise, addressing conditions that affect a narrower patient population than cardiovascular disease, diabetes, or infectious illness [5, 10]. Its methodological traditions lean towards observational cohort designs, single-centre series, and technique-oriented publications, none of which are particularly citation-dense formats [11]. The audience for any given orthopaedic paper is, almost by definition, smaller than for a paper published in a broad internal medicine journal [12, 13]. Citation half-lives in surgical disciplines are also longer, meaning the accrual process unfolds slowly, and the full impact of a paper may only become visible years after publication [14]. These features combine to produce a systematic citation disadvantage that has nothing to do with whether the science itself is well conducted [15]. What is far less clear is whether this disadvantage is stable over time or changing. The volume of peer-reviewed publications in orthopaedics has grown substantially over the past decade, subspecialisation has accelerated, and the landscape of how researchers discover and cite literature has shifted with the rise of digital indexing and open access [16–18]. Whether these trends have narrowed or widened the citation gap between orthopaedics and medicine is unknown, and the answer matters. If the disparity is growing, any evaluation system that uses raw citation data without field normalisation becomes progressively more unfair to orthopaedic researchers over time [19, 20]. A further aspect that has received little attention is the distribution of citations within each field. Science in general is governed by extreme inequality in citation accumulation, with a handful of landmark papers capturing the majority of citations while the bulk of the literature remains relatively invisible [20, 21]. Whether this concentration is more or less pronounced in orthopaedics than in medicine, and whether it has changed between 2014 and 2024, tells us something important about the internal structure of scientific impact in the two disciplines. The present study was designed to address these questions directly. By selecting the 100 most-cited articles in medicine and orthopaedics for 2 distinct publication years and comparing both absolute citation counts and time-normalised citation velocity, the aim was to quantify the magnitude of the citation gap between the two fields, determine whether that gap has widened or narrowed over the past decade, and examine whether the internal distribution of citations differs between disciplines.

## Methods

### Data source and study design

All data were obtained from the Web of Science Core Collection (Clarivate Analytics), selected for its widespread use in bibliometric research and its consistent indexing of citation relationships over time. To avoid temporal inconsistencies, citation data were extracted using a single snapshot taken on 19 January 2026. This snapshot approach ensured that all articles, irrespective of field or year of publication, were evaluated under identical conditions. Two publication years were selected a priori, 2014 and 2024. These years were chosen to represent, respectively, a mature cohort with long-term citation accrual and a recent cohort reflecting early citation dynamics in contemporary research.

### Search strategy and article identification

Articles were identified using the Advanced Search functionality of the Web of Science Core Collection. No topic-specific keywords were applied. This decision was intentional and aimed at minimising the thematic bias that could arise from keyword-based searches. Instead, records were retrieved by filtering exclusively for publication year and Web of Science subject categories. For the Medicine cohort, articles indexed under “clinical medicine” core medical subject categories were included. For the Orthopaedics cohort, only articles classified under the Web of Science category “Orthopaedics” were considered eligible. For each combination of field and publication year, results were ranked by total citation count in the Web of Science Core Collection.

### Eligibility criteria and selection of records

For each of the four predefined strata (Medicine 2014, Medicine 2024, Orthopaedics 2014, and Orthopaedics 2024), the 100 most-cited records were selected. This approach resulted in a final dataset of 400 articles with equal group sizes, facilitating direct comparison.

Only articles and reviews were included. Other document types, such as editorials, letters, meeting abstracts, and corrections, were excluded, as these formats are subject to different citation practices and would have introduced heterogeneity unrelated to scientific impact. No language restrictions were applied. Once the ranking and filters were applied in Web of Science, the selection was fully deterministic, and no manual screening or subjective judgement was required.

### Data extraction and variables of interest

For each included article, bibliographic and citation data were extracted directly from Web of Science. Extracted variables included publication year, subject category, and total number of citations recorded within the Web of Science Core Collection. Given the inherent difference in exposure time between publication cohorts, a time-normalised metric was also calculated. Citation velocity was defined as the number of Web of Science Core Collection citations accrued per year since publication. To ensure reproducibility, the elapsed time was calculated from 1 January of the publication year to the citation snapshot date.

### Outcome measures

The primary outcome of interest was the total number of citations between the four groups within the Web of Science Core Collection. The secondary outcome of interest was citation velocity, expressed as citations per year. The third outcome of interest was the distributional characteristics of citations.

### Statistical analysis

The main author (**) performed data analysis using IBM SPSS software version 25. Citation data were analysed at the article level and exhibited a markedly skewed distribution across all groups. Normality was assessed using the Shapiro-Wilk test, which confirmed a non-normal distribution of the data. Accordingly, continuous variables were summarised using medians with interquartile ranges. Between-group comparisons were performed using two-sided Mann-Whitney U tests. Comparisons were conducted both cross-sectionally, to evaluate differences between Medicine and Orthopaedics within the same publication year, and longitudinally, to assess differences between 2014 and 2024 within each field. To enable scale-independent comparisons between fields, ratios of median citation counts and citation velocity were calculated. Citation velocity was defined as the number of citations per year since publication and was obtained by normalising total citations for elapsed time. Given the descriptive and scientometric nature of the study, interpretation focused primarily on medians, interquartile ranges, and ratio-based comparisons. The exceptionally large differences observed between groups, together with the absence of meaningful overlap in interquartile ranges, were considered more informative than formal significance testing alone.

## Results

### Citation performance

Citation performance differed markedly between fields (Table 1). Articles published in Medicine demonstrated substantially higher citation counts compared with Orthopaedics in both years. In 2014, the median number of citations in Medicine was 5055 (IQR 4469–6259), compared with 288 (IQR 239.5–364.5) in Orthopaedics. A similar pattern was observed in 2024, with median values of 720.5 (IQR 567.0–1119.0) in Medicine and 34.0 (IQR 28.8–41.0) in Orthopaedics.

**Table 1:** Citation performance of the 100 most-cited Web of Science Core Collection articles in Medicine and Orthopaedics

Field	Publication year	Times cited
Medicine	2014	5055 (4469–6259)
Medicine	2024	720.5 (567.0–1119.0)
Orthopaedics	2014	288.0 (239.5–364.5)
Orthopaedics	2024	34.0 (28.8–41.0)

Data are expressed as median and interquartile range

Relative comparisons confirmed the magnitude of this disparity (Table 2). Medicine outperformed Orthopaedics by 17.6-fold in 2014 and 21.2-fold in 2024, indicating not only persistence but a further widening of the gap over time. These findings demonstrate a profound difference in absolute citation performance between the two disciplines.

**Table 2:** Relative citation performance across fields and publication years

Comparison	Median citations ratio
Medicine vs Orthopaedics (2014)	17.6 ×
Medicine vs Orthopaedics (2024)	21.2 ×
Medicine 2024 vs Medicine 2014	0.14 ×
Orthopaedics 2024 vs Orthopaedics 2014	0.12 ×

### Citation velocity

When normalised for time since publication, citation velocity remained significantly higher in Medicine than in Orthopaedics (Table 3). Median citations per year reached 419.5 (372.4–521.6) and 351.4 (276.8–544.1) for Medicine in 2014 and 2024, respectively, compared with 23.9 (19.9–30.4) and 16.6 (14.0–20.0) in Orthopaedics.

**Table 3:** Citation velocity of the 100 most-cited Web of Science Core Collection articles in Medicine and Orthopaedics

Field	Publication year	Citations per year
Medicine	2014	419.5 (372.4–521.6)
Medicine	2024	351.4 (276.8–544.1)
Orthopaedics	2014	23.9 (19.9–30.4)
Orthopaedics	2024	16.6 (14.0–20.0)

Data are expressed in median and interquartile range

Ratio-based analysis confirmed that Medicine maintained a citation velocity approximately 17–21-fold higher at both time points (Table 4). Within-field comparisons revealed distinct temporal patterns. Citation velocity in Medicine remained relatively stable over time (ratio 0.84), whereas Orthopaedics showed a reduction in the 2024 cohort (ratio 0.69), suggesting slower early citation accrual in more recent orthopaedic publications.

**Table 4:** Relative citation velocity across fields and publication years

Comparison	Median citations per year ratio
Medicine vs Orthopaedics (2014)	17.5 ×
Medicine vs Orthopaedics (2024)	21.1 ×
Medicine 2024 vs Medicine 2014	0.84 ×
Orthopaedics 2024 vs Orthopaedics 2014	0.69 ×

### Distributional characteristics of citations

**Fig. 1:**
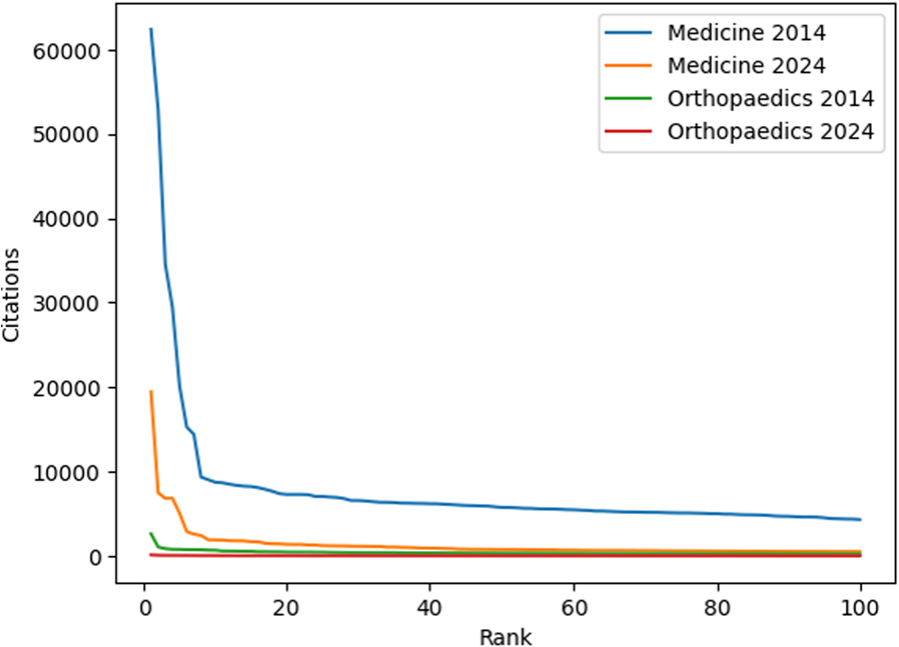
Ranked citation distributions of the Top 100 articles in Medicine and Orthopaedics (2014 and 2024)

Analysis of the full Top 100 distributions demonstrated marked differences in citation concentration between fields. In Medicine, citation counts were highly right-skewed, with a small subset of articles accounting for a disproportionate share of total citations, particularly in the 2024 cohort. In Orthopaedics, citation distributions were comparatively flatter, with a more homogeneous spread of citations across the Top 100. This difference was further reflected in lower citation counts in the highest-ranked Orthopaedic articles compared with Medicine, indicating that impact within Orthopaedics is more evenly distributed across influential publications rather than dominated by a limited number of landmark articles (Fig. 1).

## Discussion

The top-cited articles in general medicine receive roughly 17–21 more citations than the top-cited articles in orthopaedics, and this gap is actually widening. From 2014 to 2024, the median citation ratio between medicine and orthopaedics increased from 17.6- to 21.2-fold. Time-normalised analysis confirmed that this widening gap truly reflects differences in citation rates rather than the length of time articles have been available for citation.

A median of 5055 citations for the top medicine articles of 2014, compared with 288 for orthopaedics, is not a small difference that can be explained away by methodological nuances [22, 23]. It is a chasm, and the fact that citation velocity tells essentially the same story, with medicine in 2014 generating 419.5 citations per year against orthopaedics at 23.9, rules out the possibility that the gap is simply a function of time. The disparity is significant and structural; however, it does not serve as a measure of quality. This distinction follows directly from what citation counts actually measure [24]. A paper is cited when other researchers read it, find it relevant to their own work, and choose to acknowledge it [24, 25]. The size of the pool of researchers likely to do any of these things depends on how broad the topic is, how large the target patient population is, how many journals cover adjacent territory, and how many research groups worldwide are working on related questions [11, 26]. A landmark trial in cardiovascular risk prevention has a far larger potential citing audience than a similarly rigorous randomised trial comparing two fixation techniques for proximal humeral fractures ever could, regardless of how well the latter was designed and executed [5, 12].

Citation velocity in medicine between 2014 and 2024 remained broadly stable, with a ratio of 0.84 between the two cohorts, suggesting that the most impactful medical papers are accruing citations in their early years at roughly the same pace as a decade ago. In orthopaedics, the equivalent ratio was 0.69, meaning that the most-cited recent orthopaedic articles are gaining citations more slowly in their first year than their 2014 counterparts did. This deceleration could reflect increasing fragmentation within orthopaedic subspecialties, where a growing body of publications competes for the attention of a citing community that has not grown proportionally. It could also reflect changing habits in how orthopaedic surgeons engage with the literature or shifts in what types of orthopaedic papers reach the top of citation rankings.

The distributional analysis rounds out this picture in an interesting way. Medicine's citation distribution is dominated by extreme outliers: a small number of papers that attract tens of thousands of citations, pulling the overall distribution sharply to the right. In orthopaedics, the distribution is flatter, with citations spread more evenly across the top 100. This might seem like a more equitable internal structure, but it also means that orthopaedics lacks the citation-dense landmark publications that dominate aggregate metrics in medicine. There is no orthopaedic equivalent of the papers that defined modern management of sepsis, established the evidence base for statins, or described a major advance in oncological treatment, not because such advances do not exist in orthopaedics, but because the communities that would cite them are by definition smaller and more circumscribed.

The analysis was limited to the top 100 most-cited articles per field and year, a choice that ensures comparability but means that the findings describe only the extreme upper tail of each field's citation distribution. What holds for the most-cited papers may not hold for the average paper, and generalisations beyond the top 100 stratum should be made cautiously. The use of a single citation snapshot on 19 January 2026 was methodologically appropriate to ensure consistency, but it means that the 2024 cohort had been available for citation for at most 1 year, a very short window during which citation trajectories are still far from mature. Comparisons between the 2014 and 2024 cohorts must account for this asymmetry in exposure time, even though citation velocity was calculated precisely to mitigate it. The reliance on Web of Science Core Collection, while standard practice in bibliometric research, means that journals not indexed in that database are absent from the analysis, and the relative completeness of indexing may differ between medicine and orthopaedics in ways that are difficult to fully quantify. Finally, this study measured citation performance rather than research quality [27]. Level of evidence, methodological rigour, use of validated outcome measures, and relevance to clinical practice were not examined, and the data presented here say nothing directly about whether the citation gap between orthopaedics and medicine is accompanied by any difference in the intrinsic quality of the science produced.

## Conclusion

Orthopaedics demonstrated substantially lower citation performance than general medicine articles, and this gap appeared to persist over time. The observed differences likely reflect structural characteristics of disciplinary citation ecosystems rather than research quality alone. These findings suggest that citation-based metrics should be interpreted cautiously in cross-disciplinary academic evaluation.

## Data Availability

The datasets generated during and/or analysed during the current study are available throughout the manuscript.

## Declarations

### Use of AI

Language editing was performed using Grammarly (Superhuman Platform Inc., San Francisco, CA, USA).
